# Multiple Colloid-Cystoma of Left Ovary Successfully Removed

**Published:** 1889-10

**Authors:** Rudolph Menger

**Affiliations:** San Antonio


For Daniel's Texas Medical Journal.

### MULTIPLE COLLOID-CYSTOMA OF THE LEFT OVARY SUCCESSFULLY REMOVED.

BY RUDOLPH MENGER, M. D., SAN ANTONIO.

Read before the West Texas Medical Association.

I TAKE pleasure in reporting a case of successful removal of a large multiple colloid-cystoma of the left ovary, the patient rocovering.

Before going into details concerning the operation, I will briefly relate the history of the case, as it is, in some respects, quite an interesting one.

The person operated upon is a single lady, aged 49 years, of German parentage, living fourteen miles from here. Her early history reveals no particular illness, and she has had no lung, heart or any other serious sickness. Eight months ago she was treated, I am told, by a physician formerly of Comal county, for a tumor and dropsy of the abdomen, and who, after three months of fruitless treatment at San Antonio, and under promise that he could cure her entirely with medicines, wanted to operate upon her—without any assistant (!), except that he needed a nurse to take care of her; but he could not get the consent of the patient and her relatives, unless they had at least also consulted some reputable physician, at San Antonio, about the advisabilty of an operation, and procuring assistance. The latter proposition was not granted by the attendant physician, and so he discharged the case at once, and the lady was then treated at Kerrville, by a respectable physician, end of April last. There the doctor tapped ten quarts of fluid off, and in June, when the patient was again removed to San Antonio, I was cordially invited to be present at the paracentesis, when the doctor removed, with an ordinary trohcar, eight quarts of serous fluid. Before the tapping, the abdomen was very extensively distended with fluid all over the abdomen, including the left side, where a large tumor could be felt, which reached from the lower left margin of the ribs downwards to the iliac region, and about four inches toward the linea alba. From the immense loss and re-accumulation of abdominal liquid the lady was naturally a great deal emaciated. Besides this, and a sequence of mechanical pressure and obstruction of the main lower abdominal vessels, there was a great deal of œdema of the lower extremities and the back. It was with great difficulty to differentiate the tumor on the left side during the drumlike filling of the abdomen, and my collegue believed the tumor to be the left carcinomatous enlarged kidney, judging from the nodulated appearance of the tumor, the greatly emaciated condition of the patient, the appearance of blood at different times in the urine, and the œdema of back and lower extremities. The doctor told me the tumor had grown very rapidly under his observation, and, believing it to be carcinoma of the kidney, the case, of course, was pronounced as an utterly hopeless one.

After the doctor left for Kerrville, the case came into my hands. The abdomen refilled with fluid again very rapidly, and, on different subsequent examinations, also in the presence of some of our fraternity, it occurred to me that the case could not be kidney carcinoma, and free ascites and a carefully micro-urinalysis disproved any kidney disease. The urine, somewhat turbid, contained no albumen, not a trace of corpuscles and no casts or cylinder epithelium of the kidneys. I thought at first more of a tumor of the spleen, but there was no malaria or other history in the case to confirm such suspicion, and it was now settled in my mind that it must be an ovarian tumor, or rather multiple cyst, perhaps attached to a large monocyst, of which we had lately tapped the fluid; and others, who at this time examined the patient carefully, also affirmed this diagnosis. It may be allowed to mention here that the above details are given in order to show how embarrassing some cases are, and that one can't be careful enough in giving a definite conclusion as to diagnosis and prognosis in abdominal diseases, and the very best authorities have erred in this respect, and some very gravely. I may recall but a few published cases. Gaillard Thomas, for instance, in his admirable work on "Diseases of Women" says: (I quote here from the German edition, from which I translate the following:) "It is indeed not alone very difficult, but often utterly impossible, even to the most competent and experienced diagnostician, to come to a positive conclusion. There are already a large number of cases, in which experienced surgeons had not only performed laparotomy on account of their doubt concerning the nature of the tumor, but had also removed the neoplasm and the entire uterus from which it started, before they recognized the true nature of the disease. But fortunately," he says, "such things occur but rarely." "In two instances," he continues, "myself and several other consulting physicians were gravely disappointed. Just as I was in the act of extirpating a large multiple-cyst, and while the patient was already lying on the operating table, I discovered that the lady was five months advanced in pregnancy." In the other case where Thomas *suspected a very large ovarian tumor*, there escaped, on cutting through the abdominal walls, an enormous quantity of fluid, and there remained only an areola ovarian tumor, the size of a full grown head, to be extirpated.

Now, to come again to our case. The patient and her relatives were informed cautiously of the fact that there was but one way to perhaps save her from an untimely death, and that was to make an exploratory incision in order, if possible, to confirm the diagnosis, and, if advisible, to remove the entire tumor or tumors. After some difficulty she submitted to have the operation done, and that thoroughly if possible. So, this month, September 11th, I performed the operation under kind and most able assistance of Drs. A. Herff, Graham Watts, Hertzberg and Burg. As narcosis I used ether with chloroform, three parts to one. The sponges and instruments were very thoroughly cleansed and disinfected before using, and the usual incision, at an inch below the navel down to the pubic region was made. In opening the peritoneum about a quart of perfectly clear serous fluid escaped and with it, gradually the glittering bluish sack of an enormous polycyst came into view. The cyst, at sight, seemed *biacysto-sarcoma*, composed of a large number of small and larger conglomerated cystomas of gelatinous contents. The immense tumor occupied nearly the entire abdomen, being held down to the pelvic cavity by strong adhesion. In order to admit the hand freely, the abdominal incision was enlarged, and after it I had to introduce my entire arm, in order to reach and break up the adhesions which were very numerous, especially along the entire small intestines and peritoneum parietale. At the floor of the pelvis the adhesions were very difficult to break up, but gradually they were severed by the fingers and sweeping motion of the hand without instrumental aid. It was now after extending the abdominal incision about four inches upward, with much embarrassment to pull and expel the tumor out of the abdomen, even after having evacuated the sack and breaking several cysts. But gradually I succeeded, and it was found that the tumor was adherent to a narrow and quite broad pedicle which was, after some trouble, tied by the assistants with silk thread, and the tumor cut off rapidly, with a small part of its sack. The thermocauter was then used freely whenever deemed necessary. At this point of the operation there was quite severe hemorrhage at different points, and especially in Douglas' pouch, from an overlooked and bleeding vessel of the stump, but it was soon controlled by ligature, and subsequent cauterization with the thermocauter. There were still some other, but mostly parenchymatous bleeding points, and they were also soon checked by washing the abdominal cavity out with very warm water. The entire small intestines and different regions of the peritoneum abdominale showed remnants of chronic peritonitis with old and new adhesions and a coil of small intestines which was glued together with the tumor, as also a large and elongated pseudocyst with long pedicle adherent to the cyst, were both removed with the thermocauter. The uterus, bladder and ligamentum latum was free of any adhesions or degeneration and, from all we could see, every particle of the neoplasm was enu. cleated, and so we proceeded to clean peritoneal cavity. After checking all hemorrhage points, and cleaning the abdominal cavity with large quantities of pure warm water, which was done three times, the wound was closed, after also adjusting a rubber drainage tube in the excavatio recto-vesicalis or Douglas' pouch. Shortly before and after the operation was over several hypodermic injections of equal parts of ether and best brandy were made, and which had a wonderful reviving effect on her failing system. After being thoroughly cleaned and clothed and the abdominal wound dressed with iodoform cotton and salicylated cotton, a flannel bandage was applied, covering all the cotton packing well. She was then removed to a clean bed, covered with woolen blankets, and four hot water bottles were put to her body. Reaction followed very slowly, and at several times during the first day she was in a collapsed state, but by the use of hypodermic injection of ether, etc., and drinking of champagne, she slowly and radically survived the shock. About three hours after completing the operation, there was some hemorrhage oozing through the drain tube, but only very little, and it never gave any further alarm afterward. The temperature remained until the ninth day quite permanent until up to 101.5 degrees, and only on the third day it rose up to 102.5 degrees, and this was mainly atributed to an unavoidable occurrence by a faithful nurse and friend who wanted to leave her alone with the other waiters for a short time on urgent business. There were no other persons, except those appointed, allowed to enter the house during her entire after-treatment. A decided improvement of her general health was noticed, even a short time after the removal of the enormous abdominal obstruction. There was but very little nausea and vomiting after the operation, and none during the convalescing time. On the sixth day, as there was no particular indication, I removed the drain tube, which was but very slightly and partially clotted at its end with hemorrhagic and lymphatic effusion. The entire length of the abdominal incision was nine inches. The stitches were removed on the tenth day, the drain tube on the sixth day, and the wound had cicatrized by first intention.

The tumor, a multiple *colloid-cystoma* of the entire left ovary, must have weighed at least 30 pounds, as near as we could judge from the actual weight of the lacerated tumor itself, which was 15 pounds, and the loss of fluid from the sack, and gelatinous contents of some of the broken up cysts, which was also at least 15 pounds. A great deal of the gelatinous fluid, together with a number of broken up cystomas had been evacuated into the peritoneal cavity during the extirpation of the tumor. On examining the tumor at leisure I found one of the large cysts to contain an abscess cavity, about as large as an orange, situated near the middle of the tumor, and surrounded by necrosed cyst-tissue. On the outside of the tumor the enlarged left Fallopian tube was seen running in a curved line up to the margin of the cystoma. For the ligatures and closing of the abdomen only silk was used which had been put for three days in a 5 per cent. solution of carbolic acid. In regard to the narcosis, I am in general, for all other purposes, in favor of chloroform, but in a case where the system is already emaciated from chronic disease, I believe that ether, combined with chloroform, is to be preferred; in this form it acts well as an anæsthetic, and ether, by its therapeutic action as an excitant, is more apt to stimulate and maintain the nerve and heart power than chloroform alone. I am also much in favor of, and attribute a great deal of the success witnessed in some cases of other gentlemen in this city, to the free use of pure warm water for washing out the abdominal cavity, bowels, etc., before closing the abdomen. The water should have boiled be fore; and used as warm as it can safely be applied, and that in large quantities. Besides being able to remove any deleterious material, hot water stimulates the circulation and has also the tendency to control any parenchymatous or capillary hemorrhage. With it, I think, the complicated spray apparatus is avoidable, especially in private practice. Who the author of this method is, I cannot tell positively, but am quite sure it originated here in San Antonio, and, in the above case for instance, after the tumor was removed, the assistants held both abdominal walls high up, and then the abdomen was partially filled with warm water, the intestines gently washed, and then the patient was laid on one side and the water drained off again. After this the same procedure was done over, when the water became clear, and now the abdomen was sponged dry and closed, adjusting, at the same time, a drain tube. Care, of course, must be taken, in supporting the often protruding intestines and omentum while the water is evacuated. Now, gentlemen, before closing this rather extensive report, I cannot but again cordially thank all those who so ably assisted me,—the gentlemen were all hand and soul at the operation.

